# Brain morphometric similarity and flexibility

**DOI:** 10.1093/texcom/tgac024

**Published:** 2022-06-16

**Authors:** Vesna Vuksanović

**Affiliations:** Health Data Science, Swansea University Medical School, Swansea University, Data Science Building, Swansea SA2 8PP, Wales, United Kingdom

**Keywords:** morphometric similarity network, multilayer networks, flexibility, general intelligence, ageing

## Abstract

**Background:**

The cerebral cortex is represented through multiple multilayer morphometric similarity networks to study their modular structures. The approach introduces a novel way for studying brain networks' metrics across individuals, and can quantify network properties usually not revealed using conventional network analyses.

**Methods:**

A total of 8 combinations or types of morphometric similarity networks were constructed – 4 combinations of the inter-regional cortical features on 2 brain atlases. The networks' modular structures were investigated by identifying those modular interactions that stay consistent across the combinations of inter-regional morphometric features and individuals.

**Results:**

The results provide evidence of the community structures as the property of (i) cortical lobar divisions, and also as (ii) the product of different combinations of morphometric features used for the construction of the multilayer representations of the cortex. For the first time, this study has mapped out flexible and inflexible morphometric similarity hubs, and evidence has been provided about variations of the modular network topology across the multilayers with age and IQ.

**Conclusions:**

The results contribute to understanding of intra-regional characteristics in cortical interactions, which potentially can be used to map heterogeneous neurodegeneration patterns in diseased brains.

## Introduction

Cortical networks based on similarity in regional morphology estimated from anatomical magnetic resonance images (MRI) of the brain have lately been studied more extensively. So called structural covariance networks, constructed on regional cortical morphometry (e.g. gray matter volume, surface area, or its thickness) correlated across group of individuals, have provided insight into shared cortical variations of both healthy (Alexander-Bloch et al. 2013; Evans 2013; Sanabria-Diaz et al. 2010) and clinical (Bethlehem et al. 2017; Vuksanović et al. 2019) groups. Since recently, individual morphometric similarity networks (MSN) have been mapped using inter-regional associations (correlations) across the measures estimated from anatomical MRI (King and Wood 2020; Li et al. 2017; Seidlitz et al. 2018). MSNs allow for the prediction of individual differences in brain morophometry, thereby allowing for their potential utility to link brain with behaviour or other non-brain variables of interest (Doucet et al. 2019; Khundrakpam et al. 2019; Kong et al. 2018; Zhang et al. 2021) using reliable in-vivo measures of brain anatomy.

Recent studies have highlighted the potential of these networks to unveil changes in the brain during for example, normal cortical development (Galdi et al. 2020; Váša et al. 2017), dementia (Pichet Binette et al. 2020; Zhang et al. 2021), or psychiatric disorders (Morgan et al. 2019). MSNs have also allowed for relating brain physiology with behaviour, thereby allowing for an individual prediction of cognitive domains, such as general intelligence (measured by IQ) (Seidlitz et al. 2018; Solé-Casals et al. 2019; Whitaker et al. 2016), or clinical impairment (Li et al. 2017). Associations of the MSNs with non-brain derived variables have also been reported (Doucet et al. 2019).

Although the interpretation of the MSNs remains an open question, it has been found that the topology of these networks share some common properties with cortical networks extracted from other MRI modalities (Seidlitz et al. 2018). Given the potential of brain network measures to unravel large-scale cortical interactions underpinning individual differences in cognitive function and behaviour, further exploration of morphometric similarities across the cortical properties may bring about markers of the brain-cognition relationships estimated using the conventional MRI technique – anatomical, T1-weighted images. These markers have a potential clinical application in the future.

Throughout lifetime the brain undergoes complex changes in its structures that facilitate emergence of complex interactions underlying behavioural and cognitive functions. Brain ageing and cognitive function are *dynamical* processes that unfold over years on multiple levels of cortical organization in healthy and in diseased individuals. These dynamical processes are reflected in the topology of structural and functional networks (Bullmore and Sporns 2009; Gong et al. 2012; Hagmann et al. 2008).

Topological characteristics that are being linked to ageing and cognitive performance consider small-world, modular, and hub organization of the cortex as a network (Avena-Koenigsberger et al. 2018). For a modular organization, networks can be divided into modules by grouping the densely, intra-connected sub-set of nodes into a single sub-group (module or community). The brain appears to be divided into ’functional modules’ (cognitive networks) whose temporally coherent activity support cognitive function. It has been suggested that their intra-modular connectivity reflects the underlying structural (axonal) connections (Park and Friston 2013). Functional modules usually mirror local brain anatomy, however, they also incorporate long-range interactions (i.e. those between spatially distant brain areas) (Fries 2005; Vuksanović and Hövel 2014, 2015), which have only recently been linked with underlying large-scale corticocortical morphometric interactions (Vuksanović 2019; Wang et al. 2022). The evidence suggest that such interactions also map functionally similar but distributed cortical sites (Wang et al. 2022). The modular topology of brain functional networks is documented across different parcellations atlases (Bertolero et al. 2015; Meunier et al. 2009; Vuksanović 2019). Similarly, modularity as a property of brain morphology has been widely studied in the context of evolution and development (Melo et al. 2016) and studies on cortical morphometry derived from neuroimaging data suggest the modular organization of variations in regional thickness (Vuksanović 2019, Vuksanović et al. 2019), surface area (Sanabria-Diaz et al. 2010) or volume (Bassett et al. 2008). There is consistency in the organization of brain networks based on either covariations across individuals within 1 group (Sanabria-Diaz et al. 2010; Váša et al. 2017; Vuksanović 2019) or on correlations of regional characteristics at the individual brain level (Seidlitz et al. 2018). The brain modular, yet integrated, functional organization lowers the wiring cost (i.e. the average length and number of connections) of the network (Bassett et al. 2009), thus potentially lowering metabolic costs (Betzel et al. 2017) while providing more efficient information processing (Sporns and Betzel 2016). More importantly, modularity, as mapped by large-scale brain functional networks, is cognitively and behaviourally relevant; for example, it correlates with variations in performances across different cognitive tasks (Yamashita et al. 2015), greater learning ability (Bassett et al. 2011), and possibly higher intelligence (Girn et al. 2019).

However, despite of being an ubiquitous characteristic of brain topology, extracting modular structures from brain complex interactions has proved to be a difficult task, and confounded by many factors; even if as simple as the choices of regional characteristics, network resolution or brain atlas for their construction. With these confounds in mind, I sought to answer the question: Can the variations in the brain’s modular structures be better explained when analyzed using multilayer network approaches, from one individual to another, rather than by a more conventional methods of averaging across the whole group?

To answer this question, the cerebral cortex surface was for the first time, represented as a single morphometric similarity multislice network, so that its modular structures can be studied across the slices (individuals) before being averaged out. This allows for the investigations of how/which network modular interactions stay consistent across combinations of inter-regional morphometric features and individuals. This approach complements similar methodologies used in the analyses of (i) time-varying functional brain networks (Bassett et al. 2011, Braun et al. 2015, Hutchison et al. 2013, Zalesky et al. 2014) and (ii) group-wise structural covariance networks – which allow for a single cortical network construction from a group of individuals (Evans 2013, Sanabria-Diaz et al. 2010, Vuksanović et al. 2019). It should be noted, however, that the investigation of multislice community structures is not limited to the brain dynamical networks, but has been used in many other studies where network interactions take place across varying time-intervals or multivariate features (see for example (Finn et al. 2019) or (De Domenico et al. 2016)).

## Methods

### Subjects

The MRI data sets employed in this study are freely available from the public database (http://fcon_1000.projects.nitrc.org/indi/pro/nki.html), provided by Nathan Kline Institute (NKI) (Nooner et al. 2012). In total, I included 198 participants (80 female) between 6 and 83 years of age (mean: 35.5). Individuals cognitive scores in general intelligence, measured by the IQ were also included in the study. See also Fig. C.1 for age and IQ distributions across the individuals.

### Data acquisition

Anatomical T1-weighted MRI data were acquired using standard SIEMENS MAGNETOM TrioTim syngo MR B15 scanner using an MPRAGE sequence. The anatomical scan protocol is described in the following summary table (http://fcon_1000.projects.nitrc.org/indi/pro/nki/NKI_MPRAGE_PROTOCOL.pdf). Structural images were collected using a 3-dimensional high-resolution T1-weighted gradient-echo (MPRAGE) sequence [TR = 2.5 s, TE = 3.5 ms, flip angle = 8 degrees, matrix size = 256 $\times $ 256, voxel size = (1 $ \times $ 1 $ \times $ 1 mm)$^3$, 192 axial oblique slices].

### Image processing

Cortical surface reconstruction was performed using the Freesurfer image analysis pipeline, which is documented and freely available online (http://surfer.nmr.mgh.harvard.edu/); the algorithms employed for this reconstruction are discussed elsewhere (Fischl and Dale 2000, Fischl et al. 1999; 2002). Cortical morphometry was estimated from the cortical surfaces parcellated according to the 2 well-established cortical atlases implemented in FreeSurfer: Destrieux Atlas (DA) (Destrieux et al. 2010) and Desikan-Killiany Atlas (DKA) (Desikan et al. 2018; 2006). Nine properties of cortical morphometry have been estimated for each parcel (region of interest): Number of vertices, surface area, gray volume, thickness average, thickness standard deviation, mean curvature, Gaussian curvature, folding index, and curvature index. Please see also Table D.1. Imaging data were quality-controlled by visual inspection and outliers from Free Surfer output were replaced using MatLab’s (R2020b) built in function.

### Morphometric similarity networks construction

Morphometric similarity is a measure of the statistical associations between (a number of) morphometric features calculated on the reconstructed cortical surface for each cortical region. Here, I have considered 9 properties of the morphometry, which can be reliably extracted from T1-weighted images using the automated pipeline. The morphometric features were calculated for DKA (Desikan et al. 2006) and DA (Destrieux et al. 2010) cortical regions. Both brain atlases parcel the cortex based on the anatomical landmarks. DKA is a gyral based atlas (where a gyrus was defined as running between the bottoms of 2 adjacent sulci), which parcellates cortex into 34 regions per hemisphere. DA is an automated identification of classification of each vertex as sulcal or gyral, and these were then sub-parcellated into 74 labels per hemisphere. In both atlases, a gyrus only includes the cortex visible on the pial view, the hidden cortex (banks of sulci) are marked sulcus. Both parcellations have been used in a number of imaging studies in healthy and diseased cohorts.

A *N*$\times $*N* correlation matrix, representing an individual MSN, was constructed by calculating the Pearson’s correlation coefficient between the z-normalized morphometric features of region *i* and of region *j*, for all pairs (i, j) of regional properties (after replacing any existing outliers by their group median). Finally, Fisher’s transformation was employed to convert the correlation coefficients to normally distributed z-scores. This procedure resulted in either 148$ \times $148 or 68$\times $68 correlation matrices (networks) for each subject.

Four types of the MSNs were constructed: 4v-feature, 4c-feature, 5-feature, and 9-feature networks, for each of the 2 parcellations of the cortex (see also Table D.1). The 4v-feature network was constructed on the inter-regional pair-wise correlations between the volume, surface area, thickness, and thickness standard deviation. The 4c-feature networks were constructed by correlating intra-regional curvatures and their indices; 5-feature networks were construed on the intra-regional correlations between the volume, surface area, thickness, Gaussian curvature, and folding index. Finally, the 9-feature networks were constructed on all 9 features from the automated FreeSurfer’s output. Please see also Table D.1 for details. In this way, every individual was represented by 8 (4 combinations of morphometric features $\times $ 2 brain atlases) MSNs in the analysis. For more detail on the generation of the multilayer networks used in the study from the MSNs, see Bootstrapping within Statistical analysis

A fixed threshold was applied to each individual network based on the maximum spanning tree cost-function, (Gavril 1987; Li, 2022). The function preserves strongest network links (pair-wise correlations) in a way that all networks can be compared across same connection density (i.e. number of connections in the network) (Van Wijk et al. 2010). In this way, I consider only the most pronounced network links – the strongest associations in regional morphometry – which enables to ask whether these interactions (associations) are flexible or “stable/fixed” across the examined group of individuals. Using a spanning tree cost-function to threshold has been used in the context of brain networks, but only to consider nodes linked with minimum network edges (Tewarie et al. 2015).

### Multilayer network construction

To analyze nodal affiliations with their network communities across individuals, the individual MSNs were arranged in a multislice (multilayer) network, each slice being an MSN. To quantify whether the nodal affiliation with the assigned community changes across the MSNs (i.e. from slice to slice), I used the network measure flexibility. If the network flexibility shows significant variations across the slices, that would indicate that the nodal assignment to a community changes from individual to individual.

### Modularity and multilayer network modularity

For assigning nodes to their “natural network” communities a “Louvain-like” community detection algorithm can be employed (Blondel et al. 2008, Fortunato and Hric 2016). The algorithm implements a Louvain-like greedy community detection method that encodes the modularity quality index $Q$, which maximizes the partition of the network into communities (see Eq. 1). For single-slice network $Q$ is defined by summing over all matrix elements $A_{i,j}$ such that nodes $i$ and $j$ are placed in the same community. The algorithm proceeds in 2 phases (Blondel et al. 2008) repeated iteratively: quality of the partition is optimized by moving 1 node at a time until no such moves improve quality; the communities found to that point are then aggregated to build a new network where each node is assigned to a community. The output vector $S$ encodes the obtained community assignments, with $S_{i}$ identifying the community to which node $i$ has been assigned. The index $Q$ gives the quality of the resulting partition of the network. Thus, the algorithm partitions the network into communities, where nodes in a given community are highly interconnected among themselves, and sparsely interconnected to the rest of the network. The optimized modularity quality index, $Q$, was defined as follows:

(1) \begin{align*}& \textrm{Q} = \sum_{ij} [ A_{ij} - P_{ij} ] \delta(g_i,g_j) \end{align*}

where $A_{ij}$ is the strength of the edge between node $i$ and node $j$, and $P_{ij}$ is the expected weight of the edge connecting node $i$ and node $j$ under a specified null model $ P_{ij} = {k_i k_j}/{2m}$ where $k_i$ is the strength of node i, $k_j$ is the strength of node j, and $m =\frac{1}{2}\sum _{ij}A_{ij}$. The maximization of the modularity index $Q$ gives a partition of the network into modules such that the total edge weight inside of modules is as large as possible (relative to the null model), subject to the limitations of the employed computational heuristics. The Kronecker delta function $\delta (g_i, g_j$) equals one if nodes $i$ and $j$ belong to the same module ($g$), and equals zero otherwise (i.e., $\delta (g_i,g_j)$ = 1 if $g_i= g_j$ and it equals 0 otherwise). This is the simplest case, supposing that node $i$ is assigned to community $g_i$ and node $j$ is assigned to community $g_j$. The elements of the matrix $A_{ij}$ are weighted by the statistical association between regions, and I sample the distribution of partitions 1000 times to provide near-optimal $Q$ values, and then consider the network partition with the highest modularity score across these runs. The network is termed “modular” if the value of $Q$ is larger than that expected from random network null models that control for both the mean and variability of connections/correlations in the network.

Here, a multilayer community detection version of the modularity quality function was employed, which uses generalized Louvain-like community detection algorithm (Mucha et al. 2010). The algorithm allows the optimization of a modularity quality function on a network with multiple layers (slices). In this study, each individual MSN was considered being a slice/layer in multilayer networks. In the multilayer case the multislice modularity quality index, $Q_{ml}$, is given by:

(2) \begin{align*}& Q_{ml} = \frac{1}{2\mu}\sum_{ijsr} \left\{\left(A_{ijs} - \gamma_{s}\frac{k_{is}k_{js}}{2m_{s}} \right) \delta_{sr} + \delta_{ij}C_{jsr}\right\}\delta(g_{is}, g_{js}) \end{align*}

where $C_{jsr}$ is coupling or resolution parameter. That is, the conditional probability of stepping from $(j,r)$ to $(i,s)$ along slices. An inter-slice coupling is non-zero if and only if $i = j$, and it is proportional to the probability ${C_{jsr}}/{\kappa _{jr}}$ of selecting the precise inter-slice link that connects to slice $s$. $\sum _{jr}\kappa _{jr} = 2\mu $. It corresponds to the strength of the edges linking a node to itself across layers. Here, it is set to a network configuration where nodes are considered only across consecutive layers of the network. Previously, we have used network resolution of 1.1 (Vuksanović 2019, Vuksanović et al. 2019) when the DKA parcellation of the cortex was used for the division of the cortex into its natural lobe-wise communities, here, the $Q_{ml}$ utilizes re-weighting of the conditional probabilities, which allows one to work with a different network resolution $\gamma _s$ in each slice (Mucha et al. 2010). Please see Figs. D.2 and D.3 for maximum number of modules extracted for each type of the MSN across individual layers.

### Multilayer network measure: flexibility

In principle, network modular organization may vary in terms of the composition of modules (local measure) or in the number of modules in the network (global measure). Here, I focused on the composition of modules, and used flexibility $f_i$ of a node to quantify this property. Flexibility quantifies the number of times that a node changes modular assignment throughout the slices, normalized by the total number of all possible changes (i.e. by the number of consecutive pairs of layers in the multilayer framework). The flexibility $F$ of the entire network was defined as the mean flexibility over all nodes in the network:

(3) \begin{align*}& F = \frac{1}{N}\sum_{i}^{N} f_i \end{align*}

Global network flexibility was calculated by summing up the network over nodes or multislices (subjects). Different “windows-length” were used, when grouping the MSNs according to the participants’ age (into 12 age groups) or IQ (3 groups based on standard deviation from the group mean). To investigate how changes in modular assignments vary across cortical lobes, flexibility was calculated at the lobe level by dividing the cortex into 6 conventionally defined divisions: cingulate, frontal, insular, occipital, parietal, and temporal and calculating nodal flexibility averaged over the lobes.

## Statistical analysis

### Bootstrapping

To estimate the confidence interval of the flexibility values across individuals a set of 500 bootstraps with replacement was generated for each network and the cortical parcellation. That is, each statistical test was performed on the flexibility values that were averaged across 500 multilayer networks. In addition, a bootstrapping procedure was also used to calculate age-related and iq-related flexibility of the multilayers. In both cases, after bootstrapping the generated network was sorted in ascending order according to either age (for age-dependent) or IQ (for IQ-dependent) multilayers. In this way, any/if differences from an unequal step-sizes between the layers would have been cancelled out.

### Statistical tests

Since the flexibility was not normally distributed across nodes or subjects, which was established using the one sample Kolmogorov–Smirnov test, the Kruskal-Wallis (KW) statistics, a nonparametric analysis of variance test was used for statistical analyses between the groups. Results were reported as significant at the probability level *P*$<0.05$ and the corresponding group-wise statistical value for each test was also reported.

Network visualization in brain space was performed using BrainNet (Xia et al. 2013).

## Results

The main aim of this study was to investigate the modular organization of the cortex with reference to similarity in its intra-regional morphometry and “flexibility” of their interactions across multilayers. For this purpose, anatomical T1-weighted MRIs were used to extract 9 features of the cortical surface regional morphometry according to 2 well-established cortical parcellations. Correlating (a number of) combinations of the intra-regional morphometric features, 4 types of MSNs were constructed on the 2 parcellations, making it in total 8 networks per individual. Moreover, bootstraps were generated for each MSN type to establish their variations in the modular organization and the flexibility of a node across individuals. The bootstrapping was performed using 3 different setups – for randomly assigned MSNs to the network’s multislices, and for age- and for IQ-dependent bootstraps to investigate any changes in the modular structures with these 2 (brain) dynamical variables.

### Cortex as a multilayer network: global flexibility

For the investigation of the cortex as a multilayer network, an ensemble of 500 multilayer networks was generated for each of the MSNs types. This was done: (i) by randomly permuting slices to generate each multislice network, and by randomly permuting slices and then sorting these permutations by ascending (ii) age and (iii) IQ. Thus, the 3 multislice representations of the cortex for each combination of the MSNs.

**Fig. 1:**
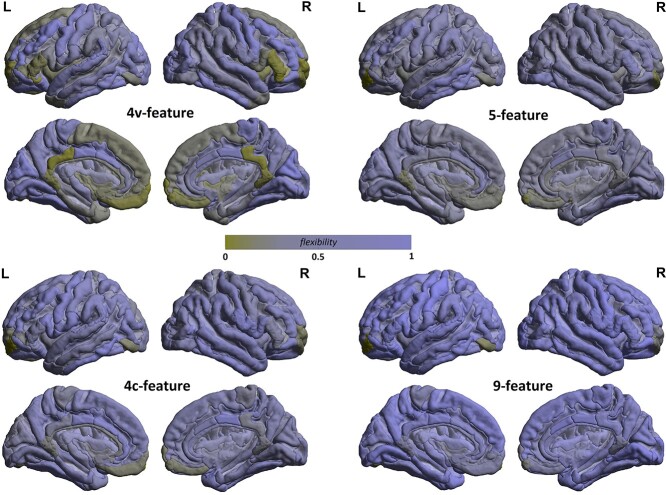
Global flexibility of the cortex as a 9-, 5-, 4v-, or 4c-feature multilayer morphometric similarity network.

Global flexibility of the 4 types of MSNs used to construct multilayers when mapped onto the cortex is shown in Fig. 1. The flexibility shown here was estimated from the bootstraps independent on subjects’ demographic or cognitive characteristics (see the above about the multislice representations). At the global network level, there was a higher flexibility of the 9-feature cortex and lower of the 4c-feature cortex ($\chi ^2$ = 649.4, $P << 0.0001$ for the comparisons between the groups). Similar results were also found for both, age- and IQ-dependent multislice networks (data not shown at the global network level, but only at the lobe level).

Some additional analyses of the flexibility values and the methodological decisions and considerations that should be taken into account for the observed similarities and differences across bootstraps and the combinations of features can be found in Appendix D. In short, differences between flexibility for the age-, IQ-dependent or independent bootstraps are shown in Fig. D.4.

It should be noted that the flexibility index of a network node can be influenced by the (optimal) number of modules that each network (a slice) was (heuristically) divided into. For the 9-feature network, a maximum number of modules was found to be 8 (see Fig. D.2), thus why usually high flexibility values for this network. However, the 5-feature network, which also re-configures into up to 8 modules, shows lower global flexibility (see Fig. D.2). This is possibly because the 5-feature network also has more nodes which interact within (only) 4 network modules, rather than 5 as in the 9f-feature network. Similarly, the 4v- and 4c-feature networks’ nodes could change affiliations across up to 5 modules. However, the 4c-feature network consistently shows a lower level of flexibility than the 4v-feature, which possibly indicates that the nodes tend to stay within the same modules. Interestingly, although almost exclusively confound within 4 modular divisions, the 4v-feature network shows either strikingly high- or low-flexibility values. These observations support the hypothesis that not only individual features, but also their (linear) combinations can reveal some additional patterns of cortical interactions that may have previously been averaged out. Please see Figs. D.2 and D.3 in the Appendix D.

### Cortex as a multilayer network: flexibility hubs

**Fig. 2:**
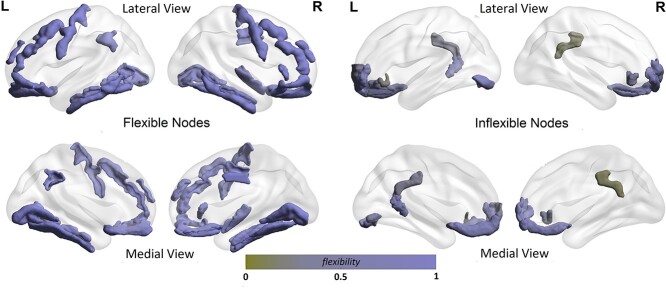
Flexibility hubs of the cortex as a 9-, 5-, 4v-, or 4c-feature multilayer morphometric similarity network: the top and bottom 5% of the nodes were selected. The top-ranked nodes represent (highly) flexible and the bottom-ranked nodes inflexible brain regions. Nodes were merged across the 4 networks. Nodal labels are given in Table D.2.

To investigate roles of cortical regions that contribute to global network flexibility the nodes were ranked to either flexible (top-ranked) or inflexible (bottom-ranked) hubs – see Fig. 2. The hubs were selected from either the top or bottom 5% (of the total number of 148 regions), for each-feature network and merged into a single list of hubs (see Table D.2). Identifying flexible and, equally important, inflexible hubs may help to understand whether/how their roles in cortical interactions underlie individual differences in cognitive performance or susceptibility to brain diseases. It has long been argued that, for example, the progression and early sites of atrophy in dementia-causing brain diseases differ across individuals. However, most studies point out that the changes could be either well localized, but heterogeneous, or distributed across the cortical surface. In this context, flexible nodes may play role in the wide-spread patterns, while the inflexible hubs may underpin focal, but heterogeneous changes. At the same time, whether the well-established cognitive (functional) networks involve flexible/inflexible hubs may also help to understand role of these networks in individual variations in cognitive performances and thereby in cognitive impairments linked to brain disorders (see also Flexibility of the cortex across the multilayers).

### Flexibility at the lobe level

**Fig. 3:**
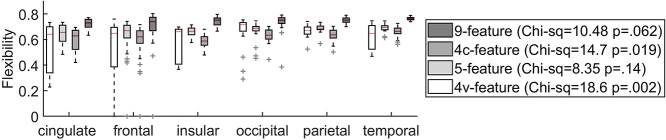
Nodal flexibility for the 4 morphometric similarity networks organized into 4 multislice networks representation of the cortex. The flexibility index was averaged over the cingulate, frontal, insular, occipital, parietal, and temporal cortex for the 9-, 5-, 4v- and 4c-feature networks. 9-feature and 4c-feature networks show higher and lower than average flexibility across the lobes. Legend shows Kruskal-Wallis test statistics for each-feature morphometric similarity network across the cortical lobes.

For the second aim, and to test the hypothesis that cortical structure underlies the patterns of corticocortical interactions at the lobe level, I assessed morphometric flexibility of the cortex across 6 cortical lobes. Each of the 3 representations of the multislice cortex was assessed using nodal flexibility averaged over 6 cortical lobes. Figure 3 show results when the randomly drown MSNs formed the multislice networks. Results for all 4 networks were shown in a single panel for easier comparisons of flexibility across different MSNs and lobar divisions. There is a degree of similarity in the behaviour of the flexibility index across the MSNs when averaged over cortical lobes. Consistent across all 3 MSN multilayers, significant differences in flexibility were found between 9- and 4c-feature networks (*P*$<0.05$), with high flexibility of the 9-feature network and lower flexibility of the 4c-feature network. Results of interests across the cortical lobes include: 1) High variability in flexibility across the cingulate, frontal and insular cortices for the 4v-feature network, where the nodes show either flexible of inflexible behaviour; 2) High flexibility, but low variability in flexibility of the occipital lobe. Statistics for these tests can be seen in the box next to the plots. Similar behaviour for age- and IQ-dependent multilayer networks and results of statistical tests can be seen in Fig. D.5.

### Flexibility of the cortical networks across the multilayers

**Fig. 4:**
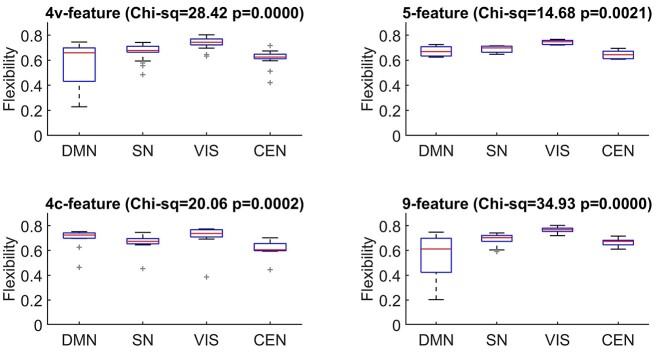
Nodal flexibility of the 9-, 5-, 4v- and 4c-feature multilayer cortex averaged over the 4 cognitive networks. Titles show Kruskal-Wallis test statistics results for differences between the networks. DMN – Default Mode Network, SN - Salience Network, VIS – Visual Network, CEN – Central Executive Network.

To test hypothesis that the previously established cortical networks supporting cognitive function display differences in flexibility of their nodes depending on the type of the multilayer network (i.e. morphometric features involved) flexibility was averaged over nodes belonging to either of the 4 cortical networks. Figure 4 shows behaviour of the Default Mode (DMN), Salience (SN), Visual (VIS), and Central Executive (CEN) Networks across different multilayers. Overall, VIS network nodes show higher flexibility than the other 3 networks. Pair-wise tests of differences after the KW test show that for the 9- and 4v-feature network the VIS has higher flexibility than all the others; for 4c-feature the VIS has higher flexibility than the DMN and SN. However, for the 4c-feature network it is the CEN network that has higher flexibility than the DMN and VIS. These results may explain some different results in the involvement of these networks across different studies. Figure D.6 shows similar results obtained when age- and IQ-dependent networks were analyzed. Interestingly, in both such multilayer networks the 5-feature cortex shows no differences when averaged over the 4 networks.

### Age- and IQ-dependent multilayer networks

**Fig. 5:**
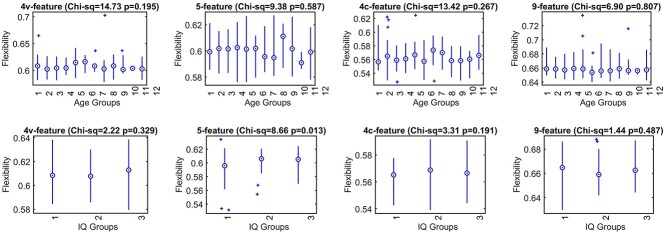
Flexibility of the 4 morphometric similarity networks across IQ-dependent (top panels) and age-dependent (bottom panels) multilayer cortex averaged over 3 IQ and 12 age groups. The 3 IQ groups are: groups 1 (iq-s.d.) and 3 (iq+s.d.), formed of subjects whose IQ was 1 standard deviation (s.d.) away from the mean and group 2 (iq-mean) within this interval. Twelve age bins (groups): group 1, 6–11 years; group 11, 60–65, and group 12, 65–85. Eleven out of 12 bins were 5 years wide. Titles show Kruskal-Wallis test statistics for each-feature network across the groups.

Figure 5 shows nodal flexibility averaged over the age- and IQ-dependent multilayers. To analyze differences in global flexibility with IQ, the flexibility was averaged over 3 IQ subgroups; groups 1 (iq-sd) and 3 (iq+sd) were formed of subjects whose IQ was 1 standard deviation (sd) away from the mean and group 2 (iq-mean) from subjects within 1 standard deviations around the mean. Since the number of subjects within groups 1 and 3 was 30 and 35 respectively, 35 subjects was randomly drawn into the iq-mean group and compared with the other 2 using a nonparametric Kruskal-Wallis ANOVA test. The iq-sd group has significantly lower flexibility compared to the other 2 groups for the 5-feature networks. Similar analysis was performed on flexibility over age-dependent multi-layers, but averaged over 12 age bins – each bin 5 years wide. Again, a nonparametric Kruskal-Wallis ANOVA test was used to test for the differences within the group. However, no significant pair-wise tests were found. It should be noted that the 9-feature networks show higher variability in flexibility across all age groups compared with other 3 networks.

### Morphometric similarity networks characteristics – positive and negative edge weights

As described in the Methods, each MSN was constructed using maximum-spanning tree cost function, to ensure that an equal number of (the strongest) links in each network enters analyses. Similar to our previous studies (Vuksanović 2019, Vuksanović et al. 2019), the network construction was done to include both positive and negative network edges. In this way, each network contains the same total number (*N* = 588) of positive and negative links, and the equal network density of 5.4% across the slices (individual networks) throughout analyses.

**Fig. 6:**
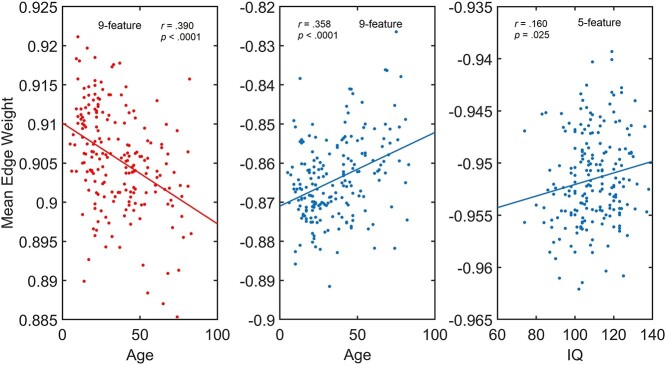
Significant correlation between positive (red) and negative (blue) edge weights averaged across individual networks with age and IQ. *r* – Pearson correlation, *P* – *P*-value.

The strength of morphometric similarity between 2 cortical regions, which was quantified as the Pearson's correlation of their regional properties, can be either positive or negative. Thus, I assessed associations of the positive and negative correlations with subjects’ age or IQ. As shown in Fig. 6, both the mean positive and negative correlation strength for 9-feature network were correlated with age and the mean negative correlation strength was correlated with IQ for the 5-feature network. Significant association between age and positive correlation strength was found also for 5-feature and 4c-feature networks and for the negative correlation strength for 4v-feature network (see Fig. D.1).

## Discussion

In this study, cortical surface morphometric similarity networks were constructed and topologically analyzed with reference to variations in the modular structures across different morphometric features. The study is first to describe the cortical surface as a multilayer network, by combining individual morphometric similarity networks into the multilayers. A total of 8 such combinations or types of morphometric similarity networks were constructed — 4 combinations of the inter-regional cortical features on 2 brain atlases. The approach introduces a novel way for studying brain networks’ metrics across individuals, and can quantify network properties usually not revealed using conventional network analyses. The results provide evidence of the community structures in MSNs as the property of cortical lobar divisions, but also as the product of different combinations of morphometric features used for the construction of the multilayer representations of the cortex.

The description of the cortex through the multiple multilayer morphometric similarity networks revealed some interesting properties of its modular structures, that could not be estimated from more conventional group-wise network analyses of its topological organization. First, using this approach I have identified (highly) flexible and inflexible morphometric similarity hubs — a characterization of the brain morphometry that could not be evaluated using standard network’s approaches. Second, by studying the nodal community affiliations at different level of cortical organization, I have characterized the variation in flexibility at the lobe and the cortical networks level. The results show that the patterns of nodal affiliations with their natural network communities are: (i) consistent across the MSN multilayers, while being dependent on (ii) number of features used to construct the multilayers and (iii) the intrinsic cortical organization into the (natural) lobar divisions. The results also show association of the flexibility with IQ and age.

In this study, 4 combinations of morphometric features were used to assess the modular organization of the cortex. The combinations were chosen based primarily on the regional or global geometry they describe and/or their mutual relationship. The multilayer cortex formed on the 9-feature MSNs shows highest flexibility both, globally and across the 6 cortical lobes. The 9-feature MSN also divides into largest number of communities (see Fig. D.2). A possible interpretation is that a larger number of network’s divisions can reveal patterns not seen in the other 3 networks, constructed on the combinations of only 4 or 5 features. For example, 4-feature networks show similarly high level of flexibility, however, their network interactions were limited to only 4 modules. Another possibility could be that the number of features determines also number of modules in an MSN; however, even though still possible, it should be noted that the 5-feature network divides into up to 8 modules. This network was constructed on similarity between the combination of intra-regional volumetric and (global) curvature measures, therefore, it is more likely that the combination, rather than the number of features plays role in the divisions.

Results at the lobe level show that the occipital cortices have higher flexibility than the other lobes for all multilayers; statistically significant for the 4c- and 4v-feature networks. Likewise, the frontal cortex shows on average lower flexibility than the occipital and temporal cortices across the 4v-feature network multilayers, while insular cortex has significantly lower flexibility compared to the parietal cortex for the 4c-feature multilayer cortex. It should be noted that the 4v-feature network displays high variability in the flexibility of the frontal, cingulate and insular regions (see Fig. 3). By closer inspection, it could be seen that the participation in modular network interactions of the frontal, cingulate and insular regions were either (highly) flexible or inflexible (please see also discussion about brain hubs and importance of inflexible interactions). It should be noted that even tough not highly varying across the lobes, the flexibility of the 4v-,4c-,5-, and 9-feature networks show similar behaviour in terms of their flexible/inflexible nodes. That is, the hubs tend to map the same regions across all the 4 multilayer networks. This can be seen in Fig. 2 that shows flexibility hubs, when merged across all the 4 multilayer networks. Majority of the flexible hubs was found in the frontal and occipital cortex and also cingulate and insular cortex. Inflexible hubs were also more frequently found among the frontal or cingulate cortices. Hubs were equally distributed across the hemispheres, however, heavily weighted towards gyri than sulci regions. There were equal number of hubs classified as gyri – 31 in total (as either being flexible or inflexible hubs) vs 23 (flexible) and 10 (inflexible) sulci hubs. Notably, the DA parcellation labels: fronto-marginal gyrus (of Wernicke) and sulcus; inferior occipital gyrus (O3) and sulcus; and transverse frontopolar gyri and sulci were repeatedly ranked as inflexible hubs across all the 4 representations of the cortical multilayers. This is an important observation, when considering results obtained for gyri-based DKA parcellation of the cortex (see also Fig. D.7).

The analysis of flexibility across the 4 well-established cognitive networks shows that the intra-regional (but also the spatial, i.e. lobar) characteristics play roles in morphometric flexibility (see Fig. 3). The VIS network shows higher than the group average flexibility across all the 4 MSN multilayers, very likely due to a relatively high portion of the occipital lobe nodes involved in this network. The DMN, even though it shows high variability in flexibility, this was again driven by its nodal lobar characteristics. That is, a high proportion of the DMN nodes are inflexible cingulate and frontal regions. Inflexible hubs, as discussed earlier, may be considered as those highly integrative regions (from the cognitive network involvement) whose brake-down has profound effect on the brain as a network (see for example (Touroutoglou and Dickerson 2019)). This may explain, for example, why the DMN is associated with a range of brain diseases (see for example (Mohan et al. 2016)). The importance of the network hubs in the context of their flexibility across the morphometric multilayers can be interpreted as a property of cortical (morphometric) plasticity, where the complex multivariate factors influence and control cortical variations dynamically (see also a discussion about possible biological considerations of these results below).

The analysis across the age- and IQ-dependent multilayer networks showed that the behaviour of the flexibility index was similar to that when the MSNs were randomly assigned to the layers. Due to the bootstrapping method applied here, differences in absolute values of the flexibility were observed between these multilayers (see Fig. D.4) but not in their behaviour across different levels of analysis (either lobar or cognitive networks). This may mean that the modular structures at the lobe level map local signals from inter-cortical features, while heterogeneous factors involved in ageing and IQ dynamics are more global brain network characteristics. This can be seen for when the flexibility was analyzed as a property of an individual network (see Fig. 5). Results show significantly lower flexibility of the cognitive group with the IQ of 1 standard deviation below the group mean (iq-sd) compared to the other 2 groups, for the 5-feature multilayer network. This difference was possibly driven by significant correlation between IQ and negative network flexibility found for the 5-feature network (Fig. D.1). Another possibility is that, similarly to the study of Sole-Casalas and colleagues (Solé-Casals et al. 2019) greater flexibility represents high versatility of the 5-feature network (see Fig. D.2). Intuitively, it would be also expected that the flexibility, like many brain network’s metrics shows changes with ageing. However, here such changes could not be detected. A several different factors may contribute to this “negative” result. As stated the above, ageing is a highly heterogeneous process with huge variability across individuals which may have influenced the results. Another possible explanation is that, unlike the negative correlation found between IQ and the 5-feature networks’ flexibility, the correlation between age and flexibility was found to be both positive and negative (Figs. 6 and D.1) for the strongest associations between the 2 (of the 9-feature network). This may mean that such associations could have cancelled out any effect of ageing when averaging flexibility across the multilayers. This observation suports the notion that positive and negative networks should be analyzed separately whenever possible.

Biologically, a leading hypothesis in the field of research into the cortical morphogenesis and plasticity suggest that the cortical morphometry (or geometry) is closely related to mechanical, geometric, and physical factors controlled predominantly by the cellular biochemistry and genetics and modulated by environmental variables (Sun and Hevner 2014, Toro and Burnod 2005). For example, regional surface area is primarily determined by the number of radial columns normal to the pial surface, and cortical thickness is determined by the horizontal layers in the cortical columns (Rakic 2009), gene expression differ between gyri or sulci (Fernández et al. 2016), thus driving differences in regional curvatures or folding. Here, a complex and rich cortical morphology, was described by a limited number (of just 9) simple measures of its geometry.

It should be also noted that, the flexibility measure used here complements prior studies that have explored nodal flexible interactions across functional brain modules extracted from fMRI data. These studies showed that the brain network nodes whose modular alliances swiftly change with the task execution, enable enhanced cognitive performances (Braun et al. 2012. 2015, Finc et al. 2020). In these studies functional networks reconfiguration has been identified as “a fundamental neurophysiological mechanisms for executive function” (Braun et al. 2015). While studies in complex structure–function relation often take into account only direct axonal links between the regions (Hagmann et al. 2008), more recent evidence has accumulated supporting the notions about the cortical functional (modular) organization being linked to the patterns of interactions between intra-regional anatomical features and cytoarchitectonic patterns (Huntenburg et al. 2018, Whitaker et al. 2016) or inter-regional morphometric networks (Vuksanović 2019). Future studies can study interplay between networks derived from multimodal MRI data. For example, informing a single MSN with the corresponding functional and/or structural network from f/dMRI data, may be an interesting way to extend this study in the quest for mapping the complex structure-function relation and a better representation of the complex cortical interactions.

## Conclusion

In conclusion, this study demonstrated how representation of the cortex in the form of a multislice network can be used to study variations in its anatomical interactions that usually stay uncovered by conventional network analyses. Using this approach, the results highlight flexible cortical regions whose network community assignments vary from individual to individual and those (inflexible) regions whose interactions are being consistent across the multilayers. The flexibility of the cortical surface multilayer may reveal individual variations in ageing and IQ, while inflexibility may map out nodal susceptibility common to brain disease.

## Supplementary Material

CerebralCortexComm_SuppInfOnly_VV_tgac024
