# Supplementary material for: Brain morphometric similarity and flexibility
Source: Cereb Cortex Commun. 2022 Jun 16;3(3):tgac024. doi: 10.1093/texcom/tgac024 (PMC9283106; doi:10.1093/texcom/tgac024)
Supplement: CerebralCortexComm_SuppInfOnly_VV_tgac024 [file cerebralcortexcomm_suppinfonly_vv_tgac024.pdf]

Supplementary Information

Brain morphometric similarity and flexibility

Vesna Vuksanovic

Demographic and Cognitive Variables

The cohort's demographic and cognitive characteristics are shown in Fig. C.1. Upper panels represent the distribution of the individuals across age and IQ and lower panels distribution of ages across three IQ sub-groups.

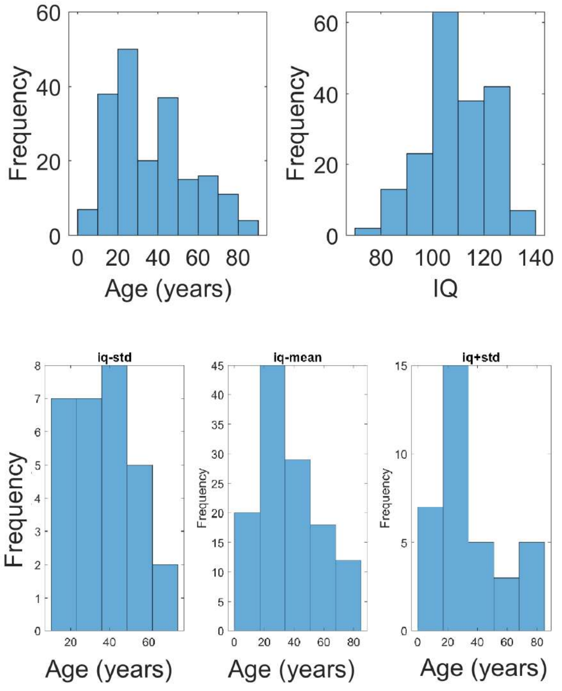

Fig. C.1. Upper Panels: Histograms showing distribution of participants' age and IQ. Lower Panels: Histograms of participants' age over three IQ groups – one standard deviation away from the group mean and within this IQ range).

Morphometric Similarity Networks Characteristics

Fig. D.7 shows nodal flexibility averaged over cortical lobes for the DKA. Similar to the flexibility across cortical lobes for the DA parcellated cortex, data for the DKA parcellation show similar patterns across combinations of features (type of multislice network). However, the results differ in terms of significant changes at the lobe level. Result of interest is lower nodal flexibility across 4v-feature networks compared to the flexibility calculated over the other three multilayer networks rather than higher flexibility of the 9-feature network. These differences can be explained either by smaller number of cortical regions over which flexibility was calculated for the DKA or this could also reflect a property of the cortex, where morphometric similarities depend on the was how the two atlases labels the cortex.

Anatomical T1-weighted images were processed and the cortex was reconstructed in subject's native space

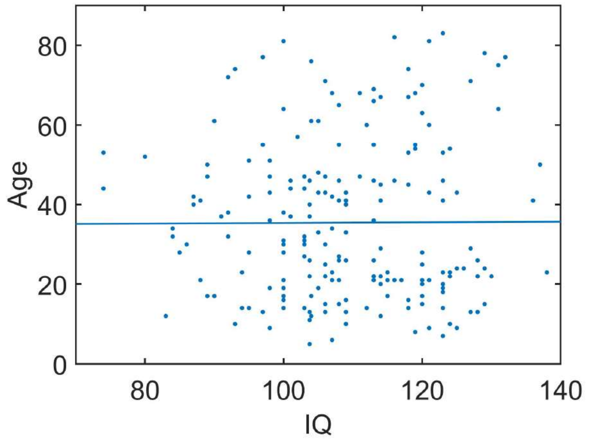

Fig. C.2. Scatter plot showing relationship between age and IQ. As expected, there is no correlation between the two variables.

using FreeSurfer (v5.3.0) pipeline. After cortical surface reconstruction nine morphometric features were extracted from regions defined by two cortical atlases the Destrieux (148 regions) or Desikan-Killiany (68 regions). Lists of regions belonging to either atlas can be found in original papers (Desikan et al., 2018; Destrieux et al., 2010). For the full list of features see Table D.1, column three. Full explanation of the

Table D.1. Morphometric features extracted from T1-weighted images used for the construction of Morphometric Similarity Networks.

4v-feature	4c-feature	5-feature	9-feature
SurfArea	MeanCurv	SurfArea	SurfArea
GrayVol	GausCurv	GrayVol	GrayVol
ThichAvg	FoldInd	ThichAvg	ThichAvg
ThickStd	CurvInd	GausCurv	ThickStd
		FoldInd	NumVert
			MeanCurv
			GausCurv
			FoldInd
			CurvInd

Abbreviations: SurfArea: Surface Area, GrayVol: Gray Volume; ThickAve: Thickness Average; ThickStd: Thickness Standard Deviation; NumVert: Number of Vertices; Mean Curv: Mean Curvature; GausCurv: Gaussian Curvature; FoldInd: Folding Index; CurvInd: Curvature Index.

nine morphometric features calculation can be found explained elsewhere, here I only briefly described each of them. The Thickness Average is an average distance between the shortest distance of a vertex on the white surface to the pial surface (not necessarily a vertex) and the corresponding vertex on the pial to the white surface (Fischl and Dale, 2000; Greve and Fischl, 2018). Surface Area is calculated as a sum of total surface area of the triangles within the region of interest. Mean curvature and Gaussian curvature were calculated using the principal (major) and minor curvatures. They have different dimensions. Folding index and curvature index were estimated based on the reconstructed surface curvatures, rather than (often used)

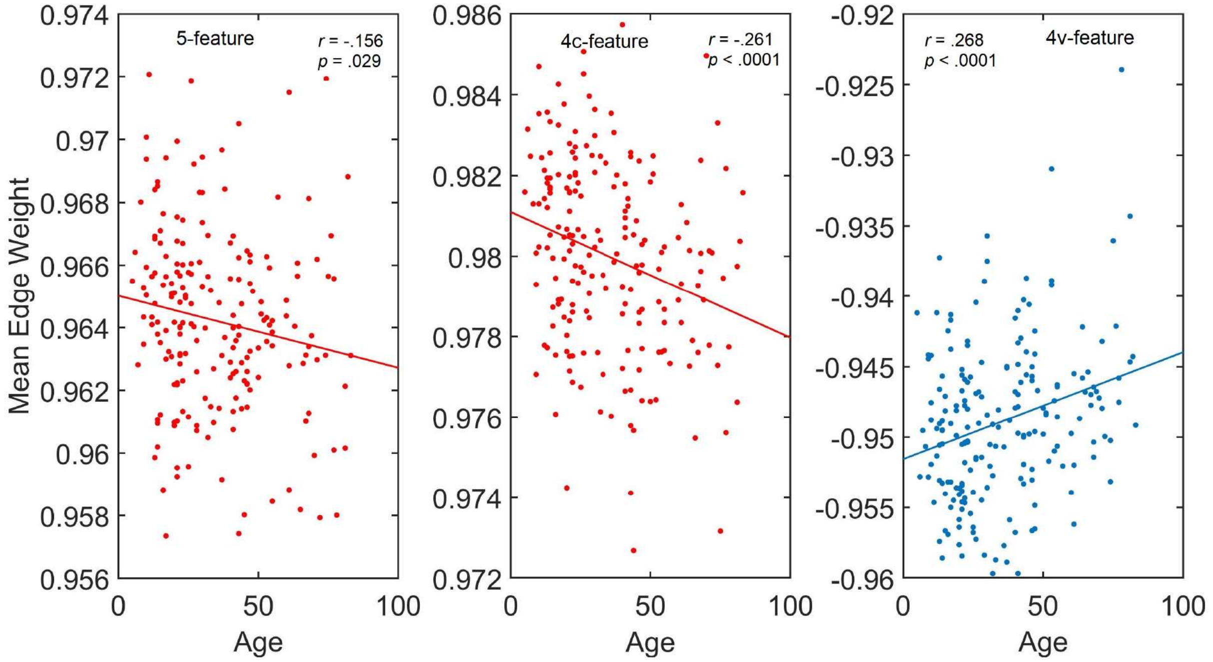

Fig. D.1. Significant correlation between positive (red) and negative (blue) edge weights averaged across individual networks with age and IQ. r – Pearson correlation, p – p-value.

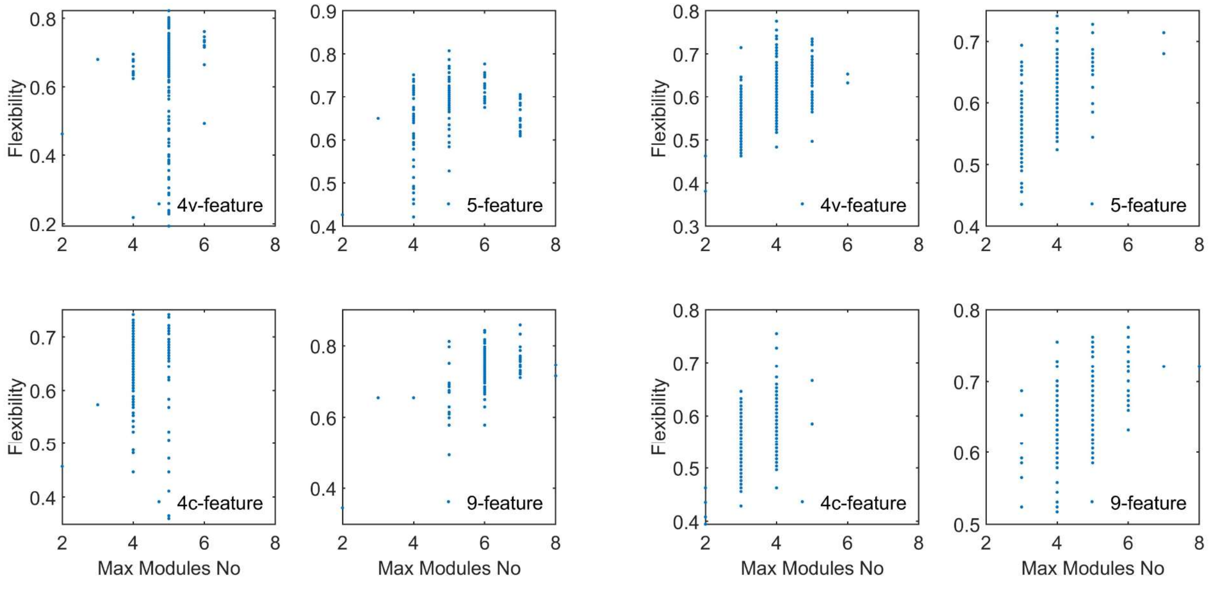

Fig. D.2. Global flexibility of the multilayers (4v-,5-,4c and 9-feature MSNs) vs maximum number of modules for that MSN averaged across network nodes (left panels) and subjects (right panels).

cortical folding (which is based on buried surfaces) (Van Essen et al., 2013).

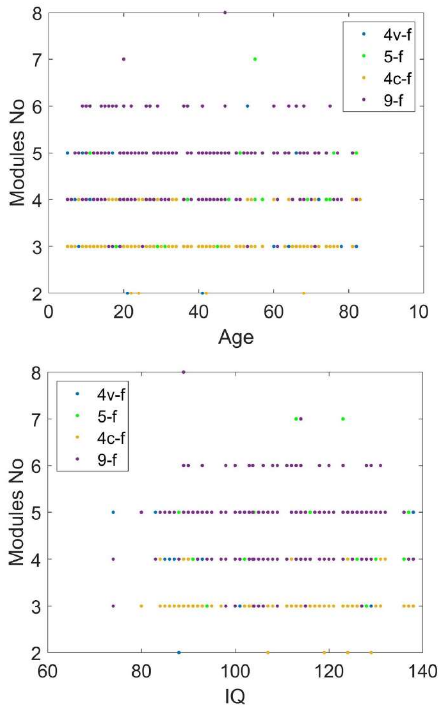

Fig. D.3. Maximum number of modules for the multilayers (4v-,5-,4c and 9-feature MSNs) vs age (left panel) and IQ (right panel).

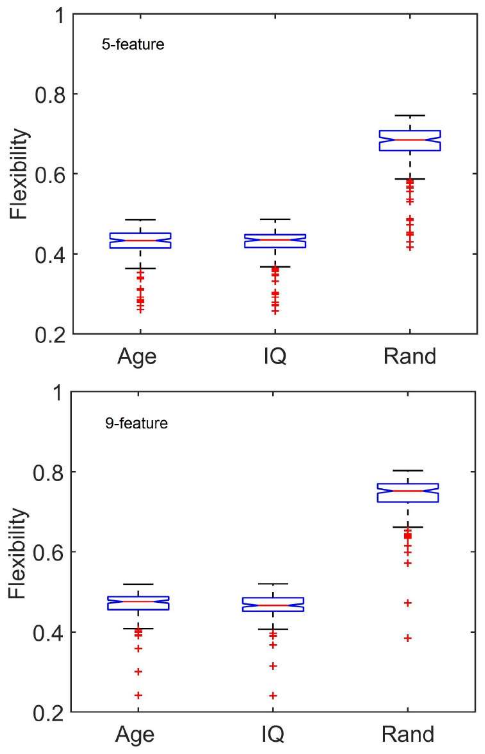

Fig. D.4. Box plots showing the mean flexibility value of age- and IQ-dependent multislices vs random assignments the MSNs to the layers. Significant differences exists for all the four networks - data exemplary shown for 9- and 5-feature MSNs.

Table D.2. Flexible cortical hubs across the multilayer MSNs. For a better visualisation of the results the hubs were merged into (highly) flexible and inflexible brain regions – a number of appearances in the list indicates how many times (the multilayers types) was ranked as a hub.

Flexible Hubs	Inflexible Hubs
lh.GandScingulAnt	lh.GandSfrontomargin
lh.GandScingul-Mid-Post	lh.GandSfrontomargin
lh.G$_{c}uneus$	lh.G$_{a}nd_{S}$ $_{f}rontomargin$
lh.Gfrontmiddle	lh.GandSfrontomargin
lh.Gfrontmiddle	lh.GandSfrontomargin
lh.Gfrontmiddle	lh.GandSfrontomargin
lh.Gfrontmiddle	lh.GandSfrontomargin
lh.Goc-templat-fusifor	lh.GandSfrontomargin
lh.Gpostcentral	lh.GandSoccipitalinf
lh.Gtempsup-Lateral	lh.GandSoccipitalinf
lh.Gtemporalinf	lh.GandSoccipitalinf
lh.Gtemporalinf	lh.GandSoccipitalinf
lh.Gtemporalinf	lh.GandSparacentral
lh.Gtemporalmiddle	lh.GandStransvfrontopol
lh.LatFis-ant-Horizont	lh.GandStransvfrontopol
lh.Scollattransvpost	lh.Gcingul-Post-dorsal
lh.Sfrontmiddle	lh.Gcingul-Post-dorsal
lh.Sfrontmiddle	lh.Gcingul-Post-ventral
lh.Soc-templat	lh.Gcingul-Post-ventral
lh.Soccipitalant	lh.Gcingul-Post-ventral
lh.Soccipitalant	lh.Gcingul-Post-ventral
lh.Sprecentral-inf-part	lh.Gfrontinf-Orbital
lh.Sprecentral-sup-part	lh.Gfrontinf-Orbital lh.Ssubparietal
lh.Grectus	
lh.Stemporalinf	lh.Grectus
lh.Stemporalsup	lh.Grectus
rh.GandScingul-Mid-Post	lh.Grectus
rh.GandScingul-Mid-Post	lh.Scollattransvpost
rh.GandSoccipitalinf	lh.Scollattransvpost
rh.GandSsubcentral	lh.Sorbital-HShaped
rh.Gcuneus	lh.Sorbital-HShaped
rh.Gfrontinf-Triangul	lh.Sorbitalmed-olfact
rh.Gfrontmiddle	lh.Sorbitalmed-olfact
rh.Gfrontmiddle	lh.Ssuborbital
rh.Gfrontmiddle	rh.GandSfrontomargin
rh.Goc-templat-fusifor	rh.GandSfrontomargin
rh.Goc-tempmed-Lingual	rh.GandSfrontomargin
rh.Gorbital	rh.GandSfrontomargin
rh.Gorbital	rh.GandSfrontomargin
rh.Gprecentral	rh.GandSfrontomargin
rh.Gprecentral	rh.GandStransvfrontopol
rh.Gprecentral	rh.GandStransvfrontopol
rh.Gtempsup-Planpolar	rh.GandStransvfrontopol
rh.LatFis-ant-Vertical	rh.GandStransvfrontopol
rh.Scircularinsulainf	rh.GandStransvfrontopol
rh.Scollattransvant	rh.GandStransvfrontopol
rh.Scollattransvant	rh.Gcingul-Post-dorsal
rh.Scollattransvant	rh.Gcingul-Post-dorsal
rh.Sintermprim-Jensen	rh.Gfrontinf-Orbital
rh.Sintermprim-Jensen	rh.Gfrontinf-Orbital
rh.Sintermprim-Jensen	rh.Grectus
rh.Soc-templat	rh.Grectus
rh.Soc-templat	rh.Grectus
rh.Soc-tempmedandLingual	rh.Sorbitalmed-olfact
rh.Soccipitalant	rh.Sorbitalmed-olfact
rh.Ssubparietal	rh.Ssuborbital

Abbreviations: lh/rh - left/right hemisphere; S-sulcus; G-gyrus.

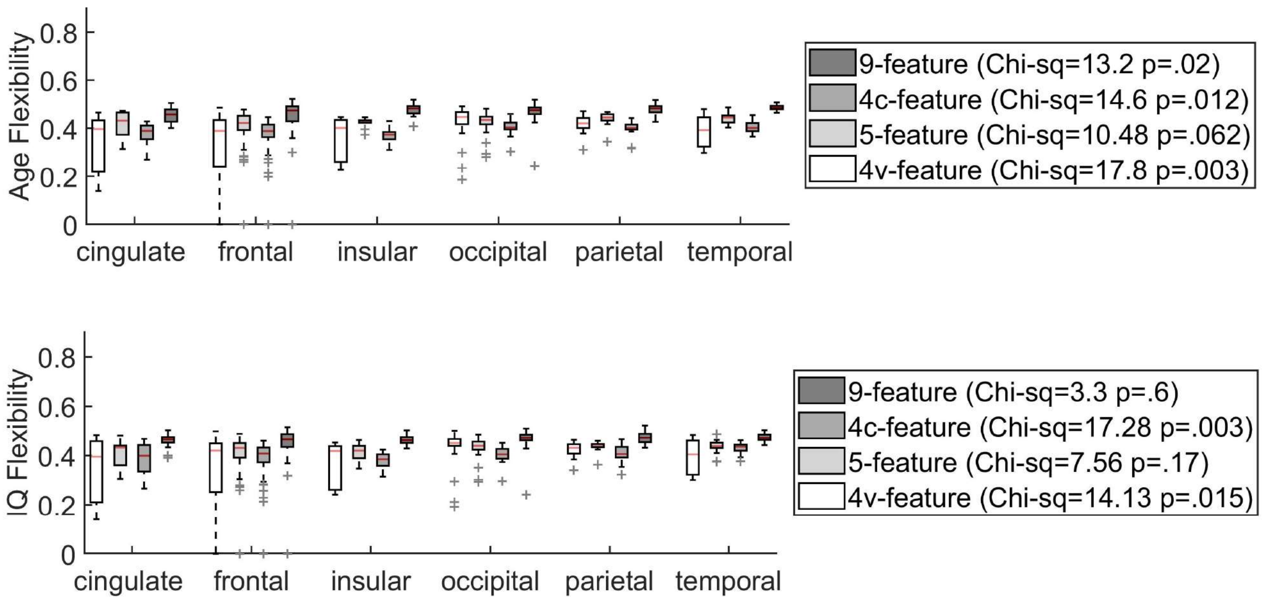

Fig. D.5. Box plots showing the flexibility averaged across age- and iq-dependent multislices for the four types of MSNs at the lobar level – the cingulate, frontal, insular, occipital, parietal and temporal cortex. Statistics shown refer to differences between the six cortical lobes for a particular type of the MSN. There are differences in lobar flexibility for the 4c- and 4v-feature networks; pair-wise similar to that of shown in Fig. 3.

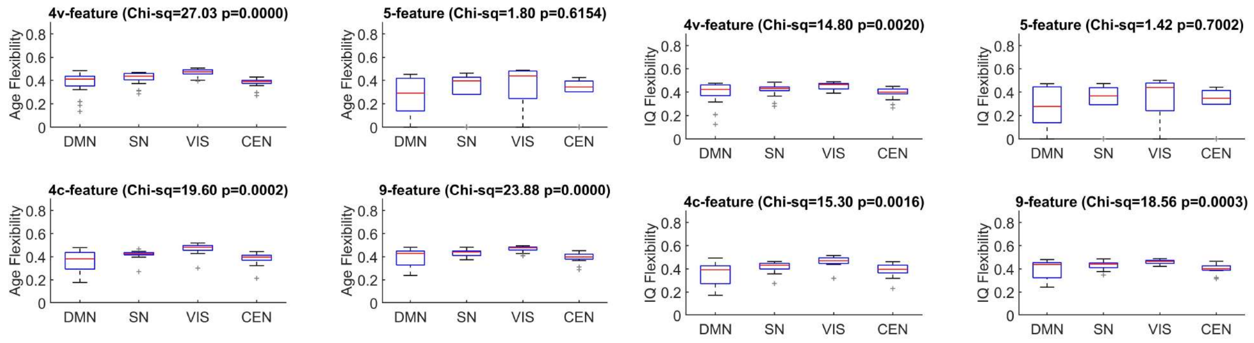

Fig. D.6. Nodal flexibility of the 4v, 5-, 4c- and 9-feature multilayer cortex averaged over the four cognitive networks for age-dependent (left panels) and IQ-dependent (right panels). Titles show Kruskal-Wallis test statistics results for differences between the networks. DMN – Default Mode Network, SN - Salience Network, VIS – Visual Network, CEN – Central Executive Network.

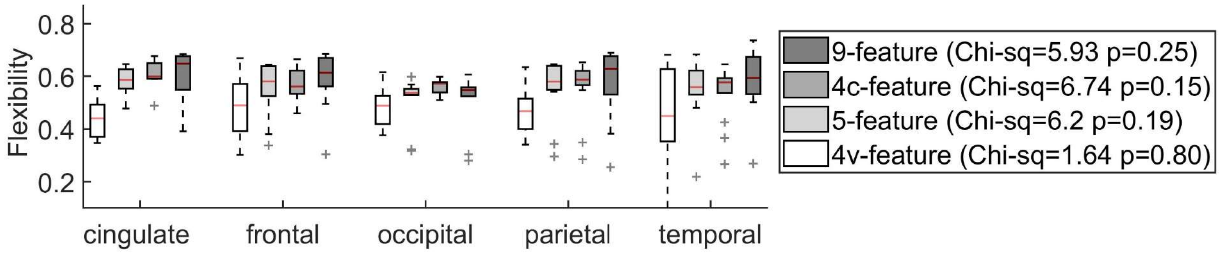

Fig. D.7. Box plots showing the flexibility averaged across multislices for the four types of MSNs at the lobar divisions – the cingulate, frontal, occipital, parietal and temporal cortex of the Desikan-Killeany Atlas. Statistics shown refer to differences between the six cortical lobes for a particular type of the MSN. There were no statistically significant differences in flexibility across cortical lobes for the DKA parcellation.
